# Exemplifying a measurement validation strategy for rare- and ultra-rare diseases: measuring what matters in alpha-mannosidosis

**DOI:** 10.1186/s13023-026-04245-1

**Published:** 2026-02-11

**Authors:** Carolyn E. Schwartz, Katrina Borowiec, Irene Koulinska, Heather Morgan, Bruce D. Rapkin

**Affiliations:** 1 DeltaQuest Foundation, Inc., 31 Mitchell Road, Concord, MA 01742 USA; 2 Departments of Medicine and Orthopaedic Surgery, Tufts University Medical School, Boston, MA USA; 3 Department of Measurement, Evaluation, Statistics, & Assessment, Boston College Lynch School of Education and Human Development, Chestnut Hill, MA USA; 4 Chiesi USA, Inc., Boston, MA USA; 5 Department of Epidemiology and Population Health, Albert Einstein College of Medicine, Bronx, NY USA

**Keywords:** Measurement, Patient-reported outcomes, Proxy report, Rare disease, Ultra-rare disease, Alpha-Mannosidosis, Lysosomal storage disorder, Caregivers

## Abstract

**Background:**

Outcomes assessment in rare and ultra-rare diseases can be hampered by the paucity of condition-specific patient-reported outcome (PRO) measures that are known to be reliable, valid, and fit for purpose. It would be helpful to develop a strategy for measuring what matters to patients/caregivers in rare and ultra-rare diseases and for validating this measurement approach even with the constraint of very small samples. The present work aims to propose a measurement strategy that builds on a conceptual measurement model of core concerns in the context of an ultra-rare lysosomal storage disorder, namely Alpha-Mannosidosis (AM). Beginning with a comprehensive literature review of what is known about AM, we identified key domains to be assessed. We drafted items for use in a video-conferenced semi-structured interview to assess symptoms in all of the relevant domains. We then identified generic, validated proxy-reported evaluative tools to assess as many of these domains as possible for use in a web-based survey. Using data collected from caregivers of individuals with AM, we then evaluated aspects of validity of the interview and survey modes of data collection.

**Results:**

The study sample included 16 AM caregivers reporting on the disease experience of 20 individuals with AM ranging in age from 4 to 49 years old. The new assessment package’s “validation” relied on a more qualitative or visual summary of the many aspects of validity, including content validity, criterion-related validity, known-groups validity, convergent and divergent validity, and longitudinal construct validity (i.e. responsiveness). It integrated information from causal (symptom measures) and effect (evaluative PRO measures) indicators to describe what quality of life concerns are linked to the AM symptom experience. Our approach emphasized effect sizes over p-values.

**Conclusions:**

This study provides a roadmap for creating meaningful outcome assessment in the context of rare and ultra-rare disease. This measurement strategy yielded an empirically-based description of the multidimensional impact of AM and characterized treatment benefits more comprehensively. The new assessment package might be used in longitudinal cohort or registry studies to capture the natural history of this ultra-rare disease, could inform future clinical trials, and capture real-world efficacy outcomes of targeted treatments.

## Introduction

Although 10% of the United States population has a rare disease^1^ [1–4], studying rare diseases remains challenging. In addition to the obvious difficulty of finding enough patients diagnosed with a rare or ultra-rare condition, there are substantial measurement challenges in monitoring disease progression or response to therapy. Outcomes assessment in rare and ultra-rare diseases can be hampered by the paucity of condition-specific patient-reported outcomes (PRO) measures that are known to be reliable, valid, and fit for purpose. The challenges in monitoring disease progression or response to therapy relate to factors such as the absence of standardized guidelines, the heterogeneity of symptoms, poor access to specialists, and poor genotype/phenotype correlations. The validation of PROs and the demonstration of a PRO being fit-for-purpose requires relatively large sample sizes that far exceed what is feasible in rare and ultra-rare disease populations. Further, utilizing generic PROs risks missing important content that captures the impact of the disease or treatment on this individual’s quality of life (QOL) and functioning and their applicability is further confounded in rare genetic disorders with considerable phenotypic heterogeneity. It would be helpful to develop a strategy for measuring what matters in rare and ultra-rare diseases and for validating this measurement approach even with the constraint of very small samples. The present work aims to propose a measurement strategy that builds on a conceptual measurement model of core concerns of an ultra-rare disease, namely Alpha-Mannosidosis (AM).

### Background on AM

**Fig. 1:**
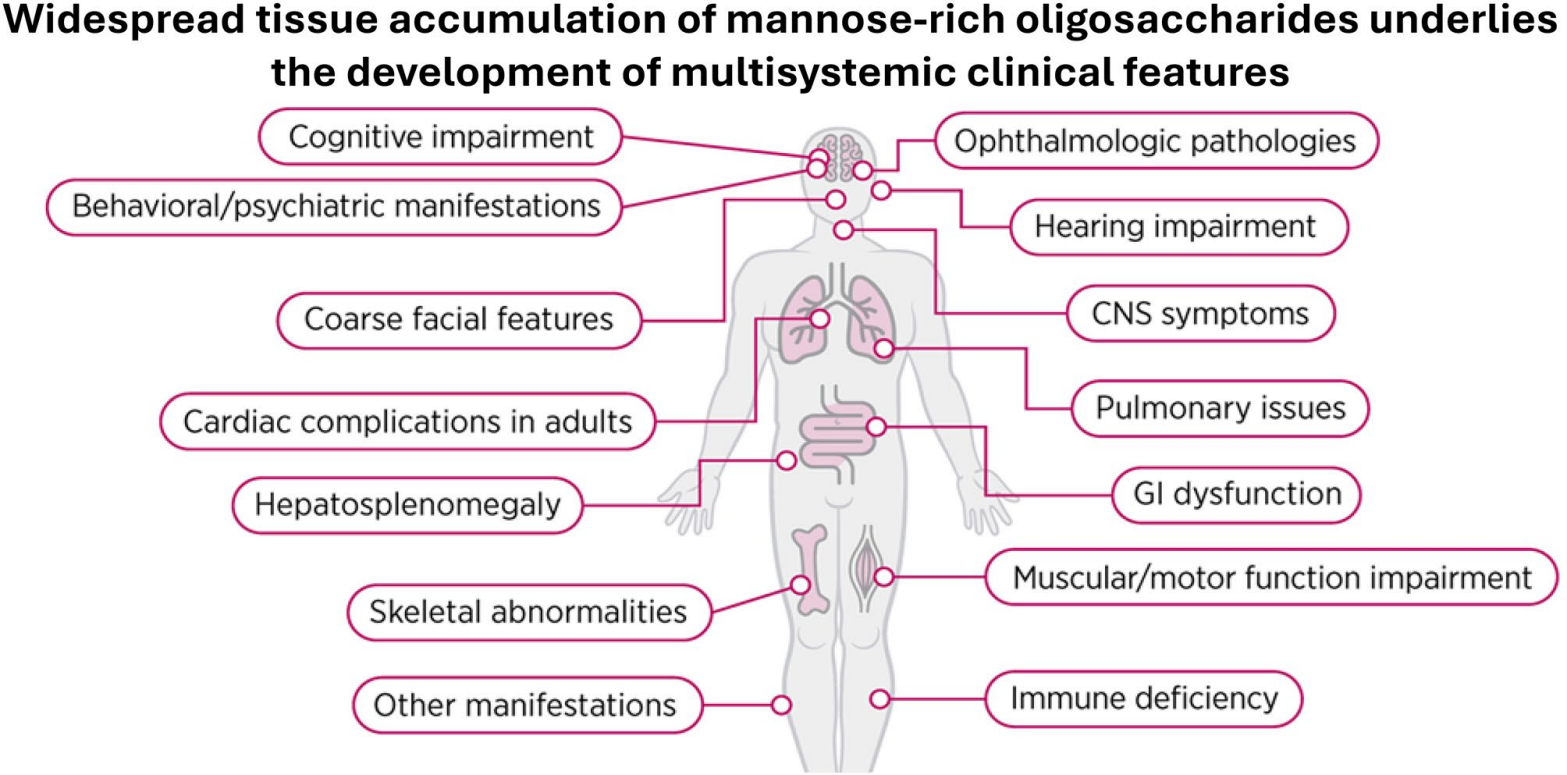
Multisystemic impact of Alpha-Mannosidosis

AM is an ultra-rare lysosomal storage disease that afflicts up to one in 500,000 live births [5], with recent estimates at one in 1,000,000 live births [6, 7]. It is caused by variants of the *MAN2B1* gene [8–10], which result in low or absent levels of alpha-mannosidase in lysosomes, leading to an accumulation of mannose-containing oligosaccharides in the cell. This accumulation affects many bodily systems and leads to a broad range of symptoms (Fig. 1).

The course of disease presentation and progression in AM is heterogenous but affected areas can include cognition, behavioral/psychiatric, coarse facial features, cardiovascular, hepatic, skeletal, ophthalmic, auditory, central nervous system (CNS), pulmonary, gastrointestinal, muscular/motor function, and immune system [5, 6, 11]. The end result of impacting multiple systems is a progressive chronic disease with heterogenous clinical presentation [5]. Manifestations can include progressive intellectual disability, hearing loss, speech impairment, difficulties with balance and walking, and frequent infections. Ophthalmologic pathologies include corneal and lens opacities, strabismus, and ocular motility disorders [5, 6, 12]. Additionally, retinal degeneration and optic nerve atrophy are increasingly recognized as significant features [5, 6, 13]. AM can lead to impaired mobility, poor sleep quality, pain, and psychological problems such as psychotic episodes, anxiety, depression, anger, and aggression/behavioral dyscontrol [6, 14, 15]. The disease can impair one’s ability to work/study and/or to be self-sufficient in such areas as eating, dressing, washing, hygiene, and other aspects of self-care [6, 14, 15]. Thus, most AM patients are reliant on caregivers and do not achieve complete independence as adults.

Treatment for AM can involve Hematopoietic Stem Cell or Bone Marrow Transplant (BMT), Enzyme Replacement Therapy (ERT), and supportive care [6]. BMT is recognized as a way to preserve neurocognitive function, prevent early death, and stabilize systemic features and cerebral function, but transplant outcomes can be variable [6, 16]. Current opinions are that BMT should be pursued as early as possible such as in the first decade of life and before the onset of significant neurological involvement. The risks associated with BMT are substantial, including graft vs. host disease, infections, endocrine disorders, pulmonary complications, and central nervous system disorders [6, 17, 18], although recent research suggests that transplant outcome and safety are improving [16]. Long-term side effects of BMT may also include secondary cancers, organ-specific complications, late infections, and QOL impairments [19]. The risk/benefit profile is thus an important consideration.

ERT is a standard of care and is the only available disease-modifying treatment for AM that has been documented to improve several biochemical and functional disease parameters [20–27]. Velmanase alfa (VA), a recombinant human lysosomal alpha-mannosidase, was approved by the European Medicines Agency in April 2018 for the treatment of non-neurological manifestations in patients with mild to moderate alpha-mannosidosis [28] and in the United States by the Federal Drug Administration in February 2023 for treatment of the non-central nervous system manifestations of AM in adult and pediatric patients [29]. VA is generally well-tolerated, although it can lead to treatment-related hypersensitivity reactions (e.g anaphylaxis) or other adverse reactions (e.g., nasopharyngitis, pyrexia, headache, and arthralgia) [22, 23, 26–29]. VA is considered in patients with parents who support the treatment, who are willing to perform regular follow-up assessments and attend the weekly treatment appointments. An integrated efficacy analysis documented that outcomes in patients receiving VA for up to four years included improved pulmonary, endurance and mobility function, and biochemical markers [22]. Real-world case studies have described improvements in immune profile, intellectual disability, speech, socialization, and QOL [30–33]. Extended long-term efficacy and safety studies indicate VA treatment benefits are maintained over a period of up to 12 years in both pediatric and adult patients with alpha-mannosidosis, suggesting that VA may successfully delay the non-central nervous system disease progression [27]. A recent caregiver survey also documented that treatment with ERT or BMT may slow the natural progression of AM [30]. Supportive care for AM may include therapies for immunodeficiency, infections, cardiac/respiratory issues, osteoporosis, and psychiatric manifestations [18]. It may also include assistive technology for hearing loss (e.g., hearing aids), mobility impairment (e.g., cane, wheelchair), occupational and physical therapy, and social care (e.g., the inclusion of a social worker or case worker to help the family navigate and coordinate care) [18]. Indeed, current recommendations for appropriate AM care include a long-term multidisciplinary care team [34].

Data are needed from robust sample sizes to document the long-term outcomes of ERT [34] and to document the AM care team and how this team changes over time.

### Objectives

Because of intellectual disability, assessment of AM symptoms and impact might best be done with proxy-reported measures (i.e., the caregiver reports on the patient’s level of functioning and symptom experience). To date, research on outcomes in individuals with AM has utilized small sample sizes and has prioritized clinical monitoring [20, 24, 26, 35] over QOL. It has relied on brief measures that yield utility scores such as the EuroQOL Five Dimension (EQ-5D) [36], and parent-reports of their child’s discomfort and impairment in self-care or activities of daily living (i.e., the Childhood Health Assessment Questionnaire [37]). While these outcomes are important, commonly used in clinical trials, and recognized by regulatory authorities, they may not reflect the full impact of AM on QOL and may miss capturing meaningful treatment benefits. The many systems affected by AM likely impact other aspects of QOL that have yet to be addressed. Further investigation into QOL profiles across domains and changes over time in these profiles are warranted.

The present work will thus present an approach for developing and validating QOL assessments in rare and ultra-rare diseases. It will demonstrate and illustrate important aspects of validity using relatively simple quantitative analysis that is viable despite small sample sizes. It will show how one can learn useful and important messages about “measuring what matters” [38, 39], even from very small samples, with clear implications for clinical research in rare diseases. This measurement strategy will enable an empirically-based description of the multidimensional impact of AM and will characterize treatment benefits more comprehensively, and thus be of direct use to clinicians, scientists, and patient-advocacy organizations. The new assessment package might be used in longitudinal cohort or registry studies to capture the natural history of this ultra-rare disease, could inform future clinical trials, and could capture real-world efficacy outcomes of targeted treatments.

## Methods

### Study population

The study participants were recruited in 2025 via collaboration with patient advocacy group registries (e.g., the International Society for Mannosidosis & Related Diseases, Coordination of Rare Diseases at Sanford), physicians treating AM patients, and panel research organizations specializing in rare patient groups (i.e., Rare Patient Voice, MedPanel). Eligible participants were English-speaking adults (age 18 or older) who are a family-member caregiver of one or more individuals with AM, are capable of providing informed consent, live in the United States, and are able to complete a video-conferenced interview and an online questionnaire. Only one caregiver per family was eligible to participate but multiple AM patients per family were accepted. The project was reviewed and approved by the WCG Independent Review Board (IRB Tracking # 20,550,752).

### Approach

Based on observations from clinical trials, ongoing registry [40], and available literature on AM natural history, we identified key domains to be assessed (Fig. 1). We drafted items (i.e., questions) for use in a video-conferenced semi-structured interview to assess symptoms in all of the domains shown in Fig. 1. We then identified generic, proxy-reported evaluative tools that were validated for non-AM specific populations to assess as many of these domains as possible for use in a web-based survey. Using data collected on caregivers of individuals with AM, we then evaluated aspects of validity of the interview and survey modes of data collection [41]. Participants were compensated for their time. The study sponsor does not have access to identifiable or individual patient information.

### Measures

#### Interview items

The interview items reflected almost all the symptoms shown in Fig. 1, with the exception being ophthalmologic pathologies^2^. Such pathologies would be expected to be captured by Other Clinical symptoms reported by the caregiver if relevant, as listing all possible such symptoms would be overly burdensome in the interview context. These Likert-scaled items ranged from 0 to 4 with higher scores reflecting worse functioning, and the option to decline to answer if the individual did not know or preferred not to answer (scored as missing). The items were rescaled from 1 to 5 to be on the same metric as the PRO scales^3^. All domains had an “Other” option that enabled the caregiver to report other symptoms within that domain that were not otherwise captured. The interview items were categorized as Activities of Daily Living (ADL), Emotional, Cognitive/Developmental, and Clinical. The seven items assessing ADL symptoms comprised aspects related to executive functioning, ability to perform household and self-care tasks, and verbal and written communication. Use of hearing aids was assessed in a separate dichotomous item. The nine items assessing Emotional symptoms comprised negative affect symptoms, autistic features, psychotic symptoms, behavioral dyscontrol, and difficulty engaging with others socially. The nine Cognitive/Developmental symptoms comprised memory problems, hyperactivity, intellectual and speech disabilities, need for a mobility assistive device, and fine and gross motor skills. The 15 Clinical symptoms comprised most of the remaining AM manifestations shown in Fig. 1, including facial features, cardiac complications, hepatosplenomegaly, skeletal abnormalities, immune deficiency, gastrointestinal dysfunction, pulmonary issues, and central nervous system symptoms.

#### Survey measures

The survey tools were drawn from the National Institutes of Health-funded Patient-Reported Outcome Measurement System (PROMIS) and Neurological Quality of Life (Neuro-QoL^TM^) item banks or short-forms assessing AM-relevant domains. These evaluative tools were selected because they are freely available to researchers, capture a broad range of content, and have been validated for the general population in large samples using item response theory methods [42, 43]. We used PROMIS or Neuro-QoL^TM^ Parent Proxy (PP)-reported item banks, in their original form or modified the self-reported item banks to be proxy-reported with permission from the developers. We utilized short-forms unless key content appeared to be missing. For example, the mobility and upper extremity short-forms were modified to include items deemed particularly relevant for AM (e.g., use of a cane or wheelchair (mobility), able to zip up clothes or cut a piece of paper with scissors (upper extremity).

The Likert-scaled items use a five-point scale (1 to 5) with higher scores reflecting better function for positively worded domains, and worse function for negatively worded domains. Respondents were “required” to answer all questions in the survey engine, so we allowed a “Not applicable/Prefer not to answer” option (coded as missing) to comply with ethical requirements to allow choice in participation.

**Fig. 2:**
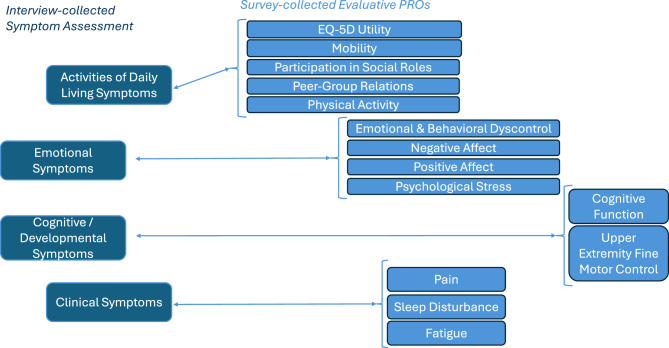
Interview and survey proxy-reported data collected

The domains assessed are shown in Fig. 2. This figure also indicates the conceptual link between the interview- and survey-collected data.

The EQ-5D-5 L Proxy Report v1 (EQ-5D) [36] was collected for validity comparisons because it is a widely used generic preference-based measure for economic modeling. This measure was designed and intended for use in cost-effectiveness and cost-utility analyses [44], and it is widely used in pharmaceutical research because of its brevity and convenience [45, 46]. It was, for example, used as measure in the VA clinical trials [24]. The proxy-report version asks the caregiver to provide their own assessment of their child’s level of impairment in five domains (mobility, self-care, usual activities, pain/discomfort, and anxiety/depression). The resulting utility score ranges from less than zero (i.e., worse than dead) to one. The United States value set was used for scoring the EQ-5D utility. Additionally, it has a visual analogue scale (VAS) item that asks the caregiver to rate their care-recipient’s health on a zero to 100 scale, where 100 means the best health imaginable and zero means the worst health imaginable [47].

### Data analysis

Because of the small sample size, the study’s validity evaluation was more qualitative than quantitative, relying on detecting patterns of associations between scores and effect sizes rather than statistical significance (i.e., p-values). Descriptive statistics of the study measures included measures of central tendency, skewness, and kurtosis. The following are the aspects of validity that were assessed.

#### Content validity

We illustrated the content overlap in the interview and survey, with the caveat that the survey assessed only a subset of the domains affected by AM because many of the features of AM are not assessed in existing PROs. We evaluated how often respondents identified symptoms that were not addressed in the initial interview protocol (i.e., “Other” symptoms mentioned by domain) and whether the interview items addressed the most important symptoms to be addressed by the treatment.

#### Criterion-related validity

We examined Pearson correlation coefficients between the interview domain scores and both the EQ-5D utility (gold standard) and EQ-5D VAS. Using linear regression models, we examined how much variance was explained in the models predicting the EQ-5D utility or EQ-5D VAS scores (dependent variables). The independent measures were either interview domain scores or PRO domain scores, and EQ-5D VAS in predicting the EQ-5D utility score. Again, the small sample size would necessarily lead to an overfit linear model so regression coefficients would not be robust, but explained variance would be a reasonable indictor of the association of content assessed in the study measures as compared to a gold standard. We focused on adjusted R-squared statistics to adjust for the number of independent variables in the model.

#### Known-groups validity

We examined mean differences by developmental age group, as AM is progressive and thus older AM patients would be expected to have worse QOL as compared to younger patients. We defined four developmental age groups: early childhood (age 4 to 9 in this sample), tweens (age 12–14 in this sample), young adults (age 20 to 26 in this sample), and middle-aged adults (age 40–49 in this sample).

#### Convergent and discriminant validity

We examined indicators of stronger vs. weaker Pearson correlations between the EQ-5D and the PRO scales, and among PRO scales to note similarities and differences in the construct(s) being measured. For example, we would expect higher correlations among measures of related constructs and lower correlations among measures of distant constructs.

#### Integrating information from causal and effect indicators

Because the interview domain scores reflected symptom profiles, they are “causal indicators” because these content areas cause worsening of QOL [48]. In contrast, items about physical, emotional, or social functioning (i.e., the evaluative PRO measures) are “effect indicators” because they reflect QOL by documenting the impact of the disease [48]. Whereas physical, emotional, or social functioning would be expected to cluster similarly across patient groups, symptoms would not. Accordingly, interpretations of the correlations of interview domain scores with the EQ-5D, the evaluative PROs, and with each other would be better interpreted as describing the AM natural history than testing an aspect of validity.

#### Possible indicators of longitudinal construct validity (responsiveness)

The data collected in this study to date are cross-sectional so we are not able to test longitudinal construct validity per se. We can, however, examine possible indicators of such by comparing PRO- and interview-scale means by four treatment groups: those who were never treated, those receiving only BMT, those receiving or having received ERT, and those receiving or having received both BMT and ERT. We would expect treated patients to do better than untreated patients, but the impact of treatment on the broad range of AM symptoms is not well understood.

*Software.* Statistical analyses were implemented using IBM SPSS version 29 [49] and Microsoft Excel.

## Results

### Sample

The study sample includes 16 AM US-based caregivers (15 mothers; 1 father), representing 20 AM patients (12 females; 8 males). Caregivers had a mean age of 54.8 (SD 14.3, range 36–78). All of the caregivers were of White race, and all had health insurance that covered their children (14 had private health insurance; 4 had Medicare, and 1 had Supplemental insurance, not mutually exclusive). Twelve of the caregivers were currently working; one was unemployed; and three were retired. Almost half of the study participants (7 of 16) had attained a master’s degree; 6 had a 4-year university degree; and the remaining caregivers had some college (*n* = 1), a technical degree (*n* = 1), or a high school diploma (*n* = 1). Sixteen caregivers reported never smoking; two were current smokers; and one was a former smoker. Caregivers reported an average of 2.6 comorbidities (SD 1.5, range 0 to 5). AM diagnosis has been documented for all 20 individuals with AM.

Table 1 provides selected descriptive statistics of the individuals with AM. Thirteen caregivers had one child with AM, two had two children with AM, and one had three children with AM. Caregivers, on average, first noticed symptoms when their child was a toddler (mean age 2.4, SD 6.6; range birth to age 30). Their child was usually diagnosed with AM in middle childhood (mean age 9.2, SD 11.7, range 1 to 43). Regarding AM treatment status, 6 AM patients had never received AM treatment, 3 had received BMT only, 7 had or currently received ERT only, and 4 had received BMT and ERT now or ever. Those receiving BMT only had completed treatment 21–23 years ago. For those receiving ERT only, treatment was ongoing and treatment duration ranged from just under half a year to just over two years. For those who had received both BMT and ERT, ERT duration was ongoing in only one of the four AM patients, with an ERT treatment duration of almost two years. For those whose ERT treatment had been discontinued, the treatment duration ranged from half a year to 3.3 years. ERT treatment was terminated primarily because health insurance would no longer pay for the treatment.

**Table 1:** Sample descriptive statistics of the people with AM

Number of Children with AM (N)	
One	13
Two	2
Three	1
Age at which caregiver first noticed AM symptoms	
Mean (SD)	2.4 (6.6)
Range	birth to 30 years old
Age at AM diagnosis	
Mean (SD)	9.2 (11.7)
Range	1 to 43
AM Individual’s Current Age	
Mean (SD)	25.6 (16.0)
Range	4–49
AM Individual’s Age Category (N)	
Early Childhood	5
Pre-teen/Tween	3
Young Adult	4
Middle-Aged Adult	8
AM Treatment Group (N)	
Never Treated	6
BMT Only	3
ERT Only	7
BMT + ERT now or ever	4

The individual interviews took an average of 79 minutes (range 44 to 120 minutes), with caregivers of one AM child taking an average of 70.7 minutes, caregivers of two AM children taking an average of 112.5 minutes, and the caregiver of 3 AM children taking 120 minutes. Surveys for the first child took an average of 51.4 minutes (range 23–146 minutes), and for the second or third child an average of 14 minutes (range 10–20 minutes). The surveys for the second or third child were shorter, because they excluded questions focused on the caregiver.

### Descriptive statistics

Table 2 provides the descriptive statistics of the study measures. Given the small sample size, it is expected that many of the distributions would be non-normal. Regarding skewness, the EQ-5D utility and the Interview Emotional Symptom scores were skewed reflecting that more of the sample was in the less-impaired ranges of the scores. Regarding kurtosis, six PP scores had negative kurtosis (PP Mobility, PP Physical Activity, PP Emotional Behavioral Dyscontrol, PP Social Participation, and PP Positive Affect), suggesting a flatter distribution with lighter tails than a normal distribution. Two scores had positive kurtosis (PP Peer-Group Relations and the Interview Emotional Symptoms score), reflecting a distribution with heavier tails and a more peaked center than a normal distribution.

**Table 2:** Descriptive statistics of interview and PRO scores

	N	Minimum	Maximum	Mean	Std. Deviation	Skewness	Kurtosis
EQ-5D Utility Score	20	−0.33	0.88	0.53	0.33	**−1.02**	0.62
EQ-5D VAS	20	0	85	56.85	23.81	−0.84	0.16
PP Mobility	20	1.25	5.00	3.29	1.23	−0.34	**−1.18**
PP Physical Activity	20	1.00	4.00	2.31	0.89	0.29	**−1.23**
PP Pain Interference	19	1.00	4.75	2.75	1.12	−0.14	−0.73
PP Sleep Disturbance	20	1.00	4.33	2.44	1.03	0.44	−0.97
PP Fatigue	20	1.00	5.00	3.07	1.03	−0.08	−0.25
PP Upper Extremity	20	1.00	5.00	3.56	1.19	−0.24	−0.73
PP Cognition	19	1.20	4.00	2.35	0.81	0.41	−0.66
PP Emotional & Behavioral Dyscontrol	20	1.43	3.88	2.52	0.79	0.13	**−1.21**
PP Social Participation	20	1.50	4.63	3.20	0.92	0.04	**−1.11**
PP Negative Affect	20	1.13	3.43	2.20	0.76	−0.14	**−1.58**
PP Peer-Group Relations	20	1.00	5.00	3.60	1.01	−0.77	**1.04**
PP Psych Stress	20	1.00	5.00	2.78	1.02	0.23	−0.18
PP Positive Affect	20	3.00	4.00	3.68	0.37	−0.67	**−1.06**
Interview ADL	20	1.43	4.86	3.06	0.97	−0.11	−0.94
Interview Emotional Sx	20	1.00	5.00	2.27	1.01	**1.08**	**1.40**
Interview Cog Dev Sx	20	1.67	4.90	3.03	0.83	0.04	−0.11
Interview Clinical Sx	20	1.27	3.69	2.37	0.68	0.01	−0.89

PP = Parent Proxy

*Note: Bolded skewness and kurtosis values are outside the acceptable range*

### Content validity

**Fig. 3:**
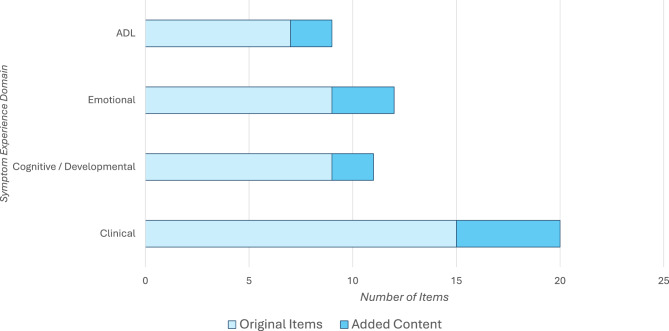
Original and added content to interview symptom experience domains

**Fig. 4:**
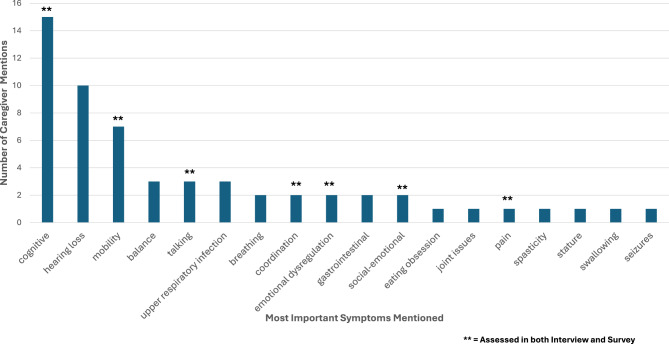
Most important symptoms and extent of coverage by interview and survey modes

Figures 3 and 4 illustrate the content validity of the assessment package. Figure 3 shows how many new symptoms were added to each of the four interview domains, with one new symptom mentioned for the ADL and Cognitive/Developmental domains, two added to the Emotional domain, and five added to the Clinical domain. Figure 4 shows the most important symptoms (*n* = 18) mentioned by caregivers in order of number of mentions (i.e., frequency) and whether these symptoms were addressed in both the interview and survey (indicated by two asterisks). When aligning what caregivers answered when asked “what are the three most important symptoms you would like solved with treatment?” with what was measured by the PROs versus the Interview, the Interview was able to provide more comprehensive information as many participants reported symptoms categorized in the “Other” item within each domain. In contrast, the generic PROs addressed only 7 of the 18 most important symptoms. It is notable that while cognition, hearing loss, and mobility were mentioned by many caregivers, there was little overlap for the remaining symptoms reported as ‘most important’ among the caregivers. For example, only 2 or 3 caregivers mentioned 8 of the most important symptoms, and only one caregiver mentioned 7. This figure highlights the heterogeneity of the AM symptom experience.

### Criterion-related validity

**Fig. 5:**
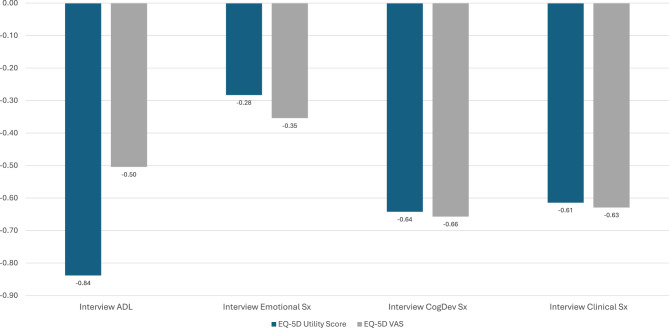
Correlation coefficients of interview scores with EQ-5D utility and VAS scores

**Fig. 6:**
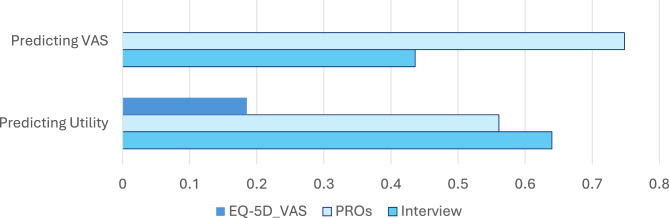
Adjusted R-squared in models predicting EQ-5D VAS and Utility score

Figures 5 and 6 illustrate the criterion-related validity of the assessment package. Figure 5 shows that the information captured in the EQ-5D utility score is largely captured by the Interview ADL domain, and relatively well captured by the Interview Cognitive/Developmental and Clinical domains (i.e., correlation coefficients ranging from −0.61 to −0.84, explaining 37–71% of the variance). The Interview Cognitive/Developmental and Clinical Symptoms domains also have similar correlations with the EQ-5D utility and VAS scores. In contrast, the Interview Emotional domain captures different information than the EQ-5D utility score (i.e., correlation coefficient of −0.28, explaining 8% of the variance), and the interview assessment of ADL and Emotional Symptoms has less overlap with the EQ-5D VAS (i.e., correlation coefficients ranging from −0.35 to −0.50, explaining 12–25% of the variance). Figure 6 illustrates the shared variance among these three types of measures. The Interview explains more variance in the EQ-5D utility scores than the EQ-5D VAS scores, whereas the PROs explain more variance in the VAS than utility scores. This pattern suggests that retaining both the interview and the survey data collection will yield useful information about both the more objective and the more subjective albeit important aspects of AM impact. For example, some of the interview items assess “objective” concerns, such as reliance on an assistive device for mobility, hearing aids, and whether the person has any of the known clinical manifestations such as coarse facial features or cardiac concerns. “Subjective” concerns are those that the clinician observer may not be privy to, such as the individual’s level of fatigue, psychosocial concerns, or the broad impact on one’s evaluation of QOL. Of note, the EQ-5D utility and VAS share relatively little variance (adjusted R^2^ = 0.185), suggesting that they are capturing distinct constructs.

### Known-groups validity

**Fig. 7:**
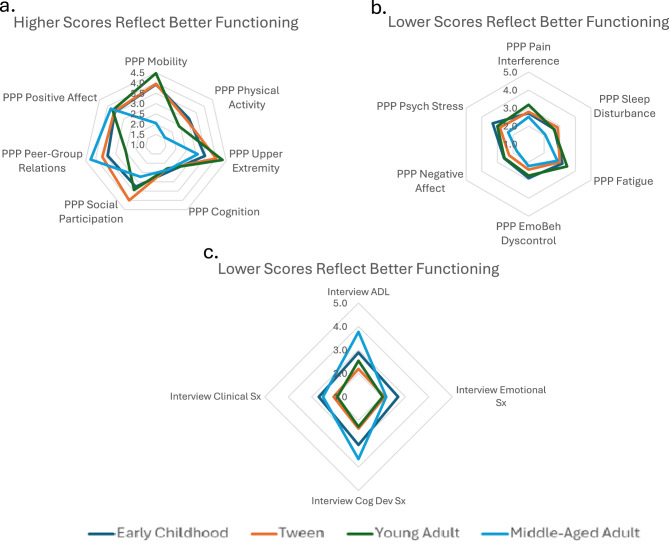
**a-c**. Radar charts of child age group differences by survey and interview outcome means

**Fig. 8:**
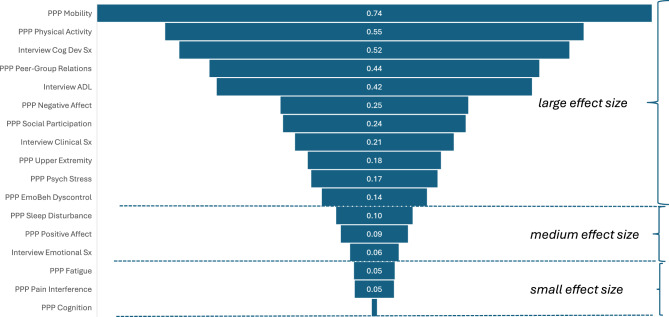
Eta-squared of child-age groups

Figures 7 and 8 illustrate known-groups validity. Figure 7 shows radar plots of mean differences by developmental age group (each group a different color line), separated by the tool’s scoring direction. Figure 7a shows PRO measures where higher scores reflect better functioning, Figure 7b for PRO measures where lower scores reflect better functioning, and Figure 7c for Interview scores where lower scores reflect better functioning. It is notable that the interview scales seem to separate the developmental age groups slightly better (i.e., the radar plot lines are somewhat distinct), with middle-aged adults functioning notably worse on ADL and Cognitive/Developmental symptoms. In contrast, the PRO measures have substantial overlap for many domains among the three groups younger than middle age. Among the PROs shown in Fig. 7b, the oldest and most disabled individuals with AM appear to have scores indicative of better functioning in the areas of pain, sleep, fatigue, emotional dyscontrol, negative affect and stress.

Figure 8 shows the eta-squared statistics for each of the linear models related to these mean scores by developmental age group. The vast majority of the outcomes considered (11 of 17) have very large effect sizes, suggesting that developmental age is important for each of outcomes and most especially for mobility, physical activity, and cognitive/developmental symptoms as assessed by the interview. Three outcomes have medium effect sizes (i.e., PP Sleep Disturbance, PP Positive Affect, and Interview Emotional Symptoms), and three have small effect sizes (i.e., PP Fatigue, PP Pain Interference, and PP Cognition). None of the outcomes have negligible effect sizes, suggesting that all of the tools used bring important information to a study of developmental age differences. This finding would support, for example, the continued use of all of the scales for a longitudinal natural history study of AM.

### Convergent and discriminant validity

Table 3 shows the Pearson correlation coefficients among the EQ-5D, PROs, and interview scales. Conditional formatting indicates effect sizes for these correlation coefficients, with more saturated colors reflecting larger effect sizes and the color corresponding to directionality. As expected, the EQ-5D utility had large effect-size correlations with PP Mobility, Upper Extremity, Cognition, and Social Participation, all of which are included in the EQ-5D dimensions. PP Mobility was most highly correlated with Physical Activity, Upper Extremity, and Social Participation. PP Sleep Disturbance had large effect-size correlations with PP Fatigue. PP Emotional/Behavioral Dyscontrol had large effect-size correlations with negative affect, psychological stress and (low) positive affect. All of the above exemplify convergent validity.

**Table 3:**
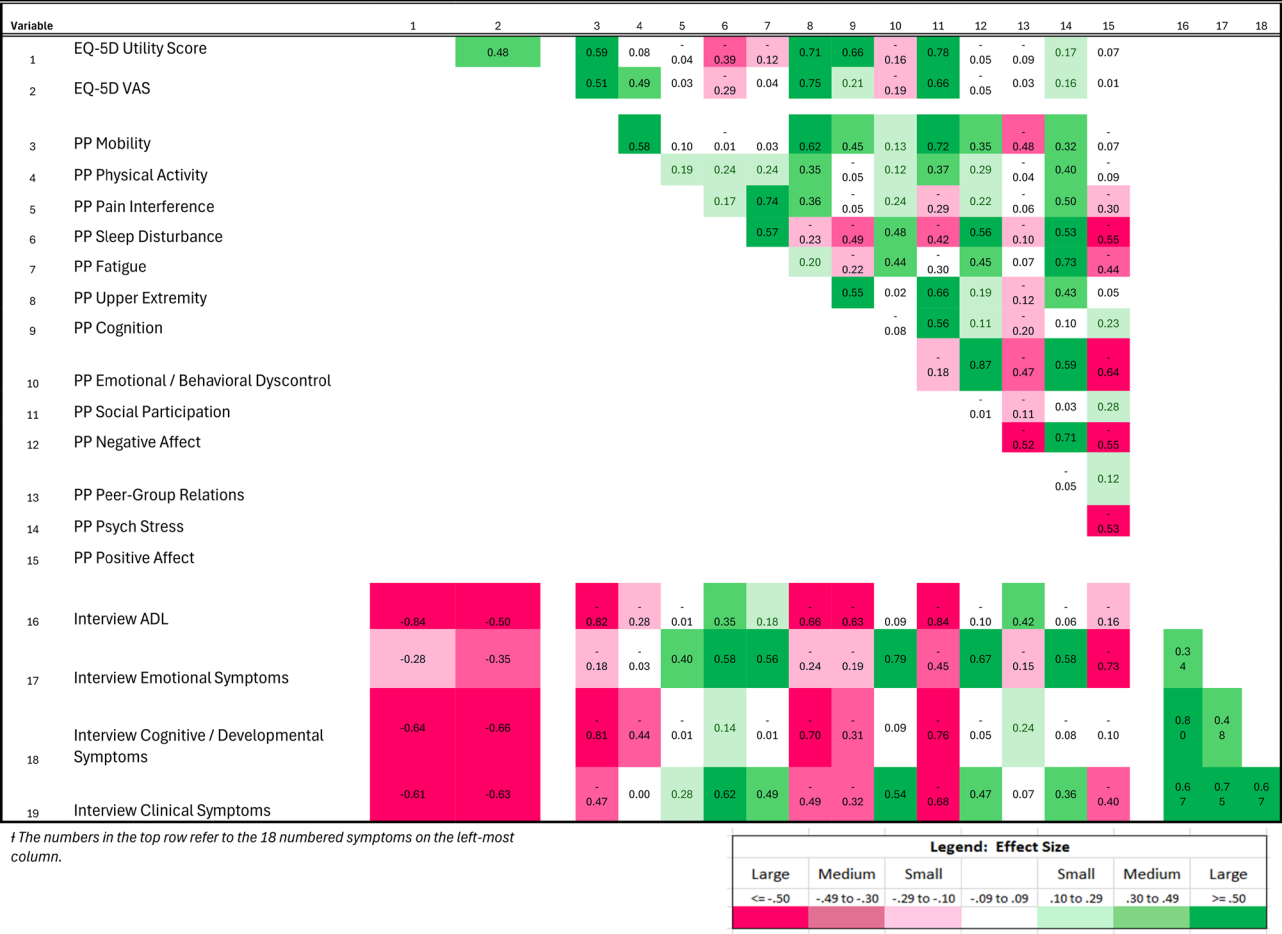
Correlation matrix of interview and PRO scoresƗ

Divergent validity is exemplified in the small effect-size correlations between PP Sleep Disturbance and PP Upper Extremity and PP Peer Group Relations, or in the negligible correlations between PP Mobility, Pain Interference, Sleep Disturbance, Fatigue, and Positive Affect.

### Integrating information from causal and effect indictors

The correlations of interview domains scores with the EQ-5D and PP scores highlight aspects of the disease not well captured by the generic PROs. While the Interview ADL, Cognitive/Developmental, and Clinical Symptoms all have large negative effect-size correlations with the EQ-5D utility and VAS scores, the Interview Emotional Symptoms score has only small or medium effect-size correlations, respectively. Thus, the emotional context of AM is not well-captured by the EQ-5D. The ADL score’s correlations with the PP scores suggests that ADL disability in AM is accompanied by mobility, upper extremity, cognition, and social participation concerns. Emotional symptoms are accompanied by sleep disturbance, fatigue, behavioral dyscontrol, negative affect, and psychological stress. Cognitive/developmental symptoms are accompanied by upper extremity and social participation concerns. Clinical symptoms are accompanied by sleep disturbance, behavioral dyscontrol, and social participation. Of note, the associations between the interview and PRO measures of cognition are only moderate effect-size correlations, suggesting that the two measures are assessing somewhat distinct constructs. An examination of the intercorrelations of the interview domains scores reveals that ADL symptoms are more closely linked to cognitive and developmental concerns and more clinical symptom burden. In contrast, emotional symptoms are somewhat less linked to cognitive/developmental symptoms, and more linked to clinical symptom burden.

### Possible indicators of longitudinal validity (responsiveness)

**Fig. 9:**
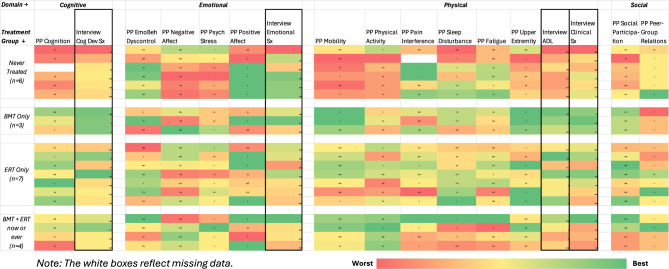
Heat map illustrating associations of treatment grouping and outcome means (red worst, green best)

Figure 9 shows a heat map of the PRO and interview scores as a function of treatment group (never treated, BMT only, ERT only, and BMT plus ERT now or ever). Conditional formatting identifies score valences with best scores shown in green through worst scores in red. To facilitate important distinctions, domains and groups are separated by a blank column or row, respectively; and interview scores are distinguished by external lines around the column. The numerical data are not shown to protect patient confidentiality because the data are at the level of the individual patient and not aggregated.

This figure illustrates that never-treated patients do worse on more domains than other groups (i.e., there are more red shaded cells for this group compared to the others). Further, patients who only received BMT appear to be doing better cognitively and physically, but worse in peer-group relations. Patients who only received ERT appear to be doing well physically, and those who are doing well physically are also doing well emotionally and socially. Some patients who only received ERT appear to be doing relatively well in symptoms related to cognition, mood, and interpersonal communication. For example, caregivers mentioned improvements in memory, performance in math classes at school, as well as hearing and speech, which was associated with being able to take part in conversations with others and interact better with their peers. Those patients who received both BMT and ERT appear to be doing worse than BMT or ERT monotherapy, but still better than those who are untreated.

When examined by domain, the cognitive/developmental interview scores and PP Cognition scores were best for those receiving only BMT, followed by either ERT monotherapy or in combination with BMT, and all are better off than untreated patients. In terms of emotional function, the negative affect PROs and interview-based emotional scores appear to tell a different story, with the latter generally reflecting better functioning than the PROs. Further, emotional dyscontrol appears to be different from both psychological stress and negative affect. In terms of the physical domain, the ADL and Clinical symptom interview scores were best for those receiving only BMT, followed by either ERT monotherapy or in combination with BMT, and all are better off than untreated patients. In the social domains, the two PP measures tell different stories, with social participation looking best for BMT-only followed by ERT-only, then BMT + ERT therapy, and the untreated looking worst off. In contrast, in peer-group relations, the untreated patients look best off, followed by BMT + ERT therapy, then ERT-only, and finally BMT-only being worst off.

## Discussion

This study provides a roadmap for creating meaningful outcome assessments in the context of rare and ultra-rare diseases. Beginning with a conceptual model that builds on what is known about the condition, it guides the selection of existing generic item banks or short forms in publicly available instruments. Subsequent item writing is done to capture all the other aspects of the known symptomatology of the condition. These items are implemented in the context of a video-conferenced interview that allows and encourages caregivers to add missed symptoms so the description of their child’s illness experience is fully described. The “validation” of the new assessment package then relies on a more qualitative or visual summary of the many aspects of validity, emphasizing effect sizes over p-values even when descriptive or inferential statistics are calculated. This roadmap thus aims to approximate validity testing despite very small samples.

The descriptive analysis of outcomes as a function of treatment group may help to elucidate AM patient experience. For example, the finding that patients who only received BMT appear to be doing better cognitively and physically, but worse in peer-group relations, may be due to the social isolation imposed by the BMT regimen to prevent infections. Patients on BMT may be required to self-isolate for a year or more to prevent infections [50]. This social isolation can be a source of suffering [51], emotional struggles and feeling overwhelmed [52].

It was interesting to note the counter-intuitive findings in Fig. 7b: the oldest and most disabled individuals with AM appeared to be better off in terms of scores on pain, sleep, fatigue, emotional dyscontrol, negative affect and stress. It is possible that these findings may reflect adaptation effects (i.e., response shift [53, 54]). In other words, either patients, caregivers, or both parties may have changed their internal standards, values, or conceptualization of the symptom being measured over the many years of this progressive and disabling condition. Changes in internal standards and values might be exemplified by patients prioritizing symptoms differently based on their disease severity. For example, sleep problems, fatigue, and stress might only be noted when the patients get better from something worse, such as difficulty breathing from decompensated heart failure. But if the patient is experiencing other severe symptoms, they might not report sleep problems, fatigue or stress because they ignore them and focus on the most bothersome other symptoms at the moment. It does not mean they have less severe sleep problems, fatigue, or stress, but rather that they have bigger problems. Response-shift effects have been noted in many medically ill patient populations and caregivers and are important when considering resilience in difficult situations [55–61]. They are also pertinent to treatment effects, and research has documented that when response shift effects are considered (i.e., adjusted for) in clinical trials data, the treatment benefits are often larger and clinically meaningful [62].

It is also notable that the PP cognition scores look different (mostly red/orange) from the Interview Cognitive/Developmental Symptoms (mostly yellow), particularly in the untreated group. These two measurement approaches differ somewhat in content. The PP cognition scores tap applied aspects of cognition, such as the parent’s evaluation of their child’s reaction time when playing with others, spatial memory, sustained attention, reading comprehension, ability to follow pictorial instructions, ability to do addition or subtraction in their head, dependence on lists to remember things, word finding, and name recall. In contrast, the Cognitive/Developmental symptoms assessed in the interview contain a mix of cognitive and developmental disabilities: memory problems, hyperactivity, intellectual and speech disabilities, need for a mobility assistive device, and fine and gross motor skills. Future research might further develop the Cognitive/Developmental symptoms addressed in the interview, perhaps separating them into two distinct scores rather than one combined score, and perhaps expanding the cognitive symptoms addressed.

The limitations of the present work should be acknowledged. First, the very small sample size limits the analyses to illustrative figures. Even descriptive statistics (e.g., means) or simple correlational analyses would be overly influenced by outliers. The descriptive analysis of treatment-group differences is similarly limited by the small sample size. For example, with only four patients in the BMT + ERT therapy group, the heterogeneous outcomes scores across the investigated domains could be reflective of differences in disease severity and age, and thus level of disability at treatment start, treatment duration, insurance coverage of other supportive therapies, or other patient factors. Second, ceiling/floor effects may have affected the group-comparison illustrations, accounting for the significant overlap in some of the child-age groups shown in the radar plots. Third, parent-proxy data are limited to the extent that they rely on the parent’s ability to pick up on cues as to how their child is doing, which may be particularly challenging if their child is very young and nonverbal, or older but severely cognitively disabled. Further, we adapted items to be proxy-reported as necessary, with the developer’s permission. While changing the reporter from self- to proxy report is unlikely to alter the measurement properties of the items, proxy report is generally considered different from patient report in terms of the perspective reflected and biases toward worse QOL compared to patients’ self-report [63]. Since most AM patients are unlikely to be able to self-report due to cognitive disabilities, using proxy-report to estimate AM patient functioning and QOL is considered the best option. Future research should continue the iterative process of psychometric validation of these tools when sufficient sample sizes are available. Fourth, missing data resulted from caregivers not knowing how to address questions when their person with AM did not have one or more concerns noted in the various PRO measures. For example, if they do not have friends, how does one answer questions about peer relations? If they do not have pain, how does one answer how pain interferes with their functioning? To reduce the extent of missing data, we reached out to all study participants during the data management phase of the present work to ask them to choose one of the answers so their child’s data did not get dropped from the analysis. This issue is, however, likely a challenge for other constructs and medical conditions. It would be worthwhile to mention the impact of answering “Do not know/prefer not to answer” at the end of the interview (and thus prior to the survey) to avoid missing data. Further, the interview domain “scores” are not fully psychometrically validated, unidimensional scores like the PRO scores. Thus, symptom items grouped in one domain may belong better in another domain based on quantitative analysis but would require larger sample sizes to implement the pertinent psychometric tests to determine where they best belong, such as factor analysis. Future work might implement factor analysis on the symptom items in a larger sample, i.e., at least ten respondents per item in a given domain [64]. Future work with this interview-based measure might also improve items by adding content reflective of what was deemed missed by caregivers and derive a way to integrate the dichotomous “hearing aid” item into one of the domains. As measurement development is an iterative process, such modifications are to be expected. Finally, the heat-map findings regarding treatment-group outcomes of BMT-only as compared to the other groups could simply be a function of earlier intervention with BMT. Most of the enrolled patients on ERT are older and would not have started ERT at the same age as BMT was given. Thus, the different age of treatment intervention is a limitation in interpreting these findings. Future clinical research might focus on AM children who started ERT at a comparable age to receiving a BMT.

## Conclusions

The measurement strategy illustrated in the present work yielded an AM conceptual model that captured relevant information, has good initial indicators of content, criterion-related, known groups, convergent/divergent, and (pseudo) longitudinal construct validity. It yielded an empirically-based description of the AM disease experience. Current state-of-the-art methods often seen in rare diseases research typically use a tool that was originally intended for population-level resource allocation decisions (i.e., the EQ-5D). In comparison, the content captured by this assessment approach is likely to better describe the multidimensional impact of the disease both cross-sectionally and over time, and to characterize treatment benefits more comprehensively. The new assessment package might be used in longitudinal cohort or registry studies to capture the natural history of this ultra-rare disease and might inform clinical trial design and real-world data collection on treatment effects. It is our hope that this new assessment package, with its more comprehensive assessment of meaningful outcomes, will be used by clinicians, scientists, and patient-advocacy organizations to shape their work with and among patients affected by AM.

## Data Availability

The study data are confidential and thus not able to be shared.

## Abbreviations

ADL: Activities of Daily Living
AM: Alpha-Mannosidosis
BMT: Bone marrow transplant
CNS: Central Nervous System
ERT: Enzyme replacement therapy
EQ-5D: EuroQOL Five Dimension Five Levels (EQ-5D-5 L) Proxy Report v1
PROMIS: Patient-Reported Outcome Information System
PP: Parent Proxy
PRO: Patient-reported outcome
QOL: Quality of life
VA: Velmanase alfa
VAS: Visual analogue scale

## Footnotes

1 A rare disease is defined as affecting fewer than 200,000 people in the United States or fewer than 65 per 100,000 per the World Health Organization. An ultra-rare disease is defined as affecting fewer than 1 in 50,000 people.

2 For full item content, please contact the first author.

3 The PROMIS/NeuroQOL items are on a 1 to 5 scale. The scale scores may be done as T-scores if the respondent population is similar to the group on which the scores were calibrated. In AM, this is not the case, so at this stage (i.e., when we do not have a sufficient sample size to do the IRT analyses to confirm model fit), we are using simple item means within a subscale as a way of describing the study sample. We have also verified that our scores are in the same direction as the PROMIS/NeuroQOL scales, such that higher scores correspond to a higher level of the construct of interest.
